# Supplementary figures and images for: COEXIST: Coordinated single-cell integration of serial multiplexed tissue images
Source: PLoS Comput Biol. 2025 Aug 5;21(8):e1013325. doi: 10.1371/journal.pcbi.1013325 (PMC12338771; doi:10.1371/journal.pcbi.1013325)

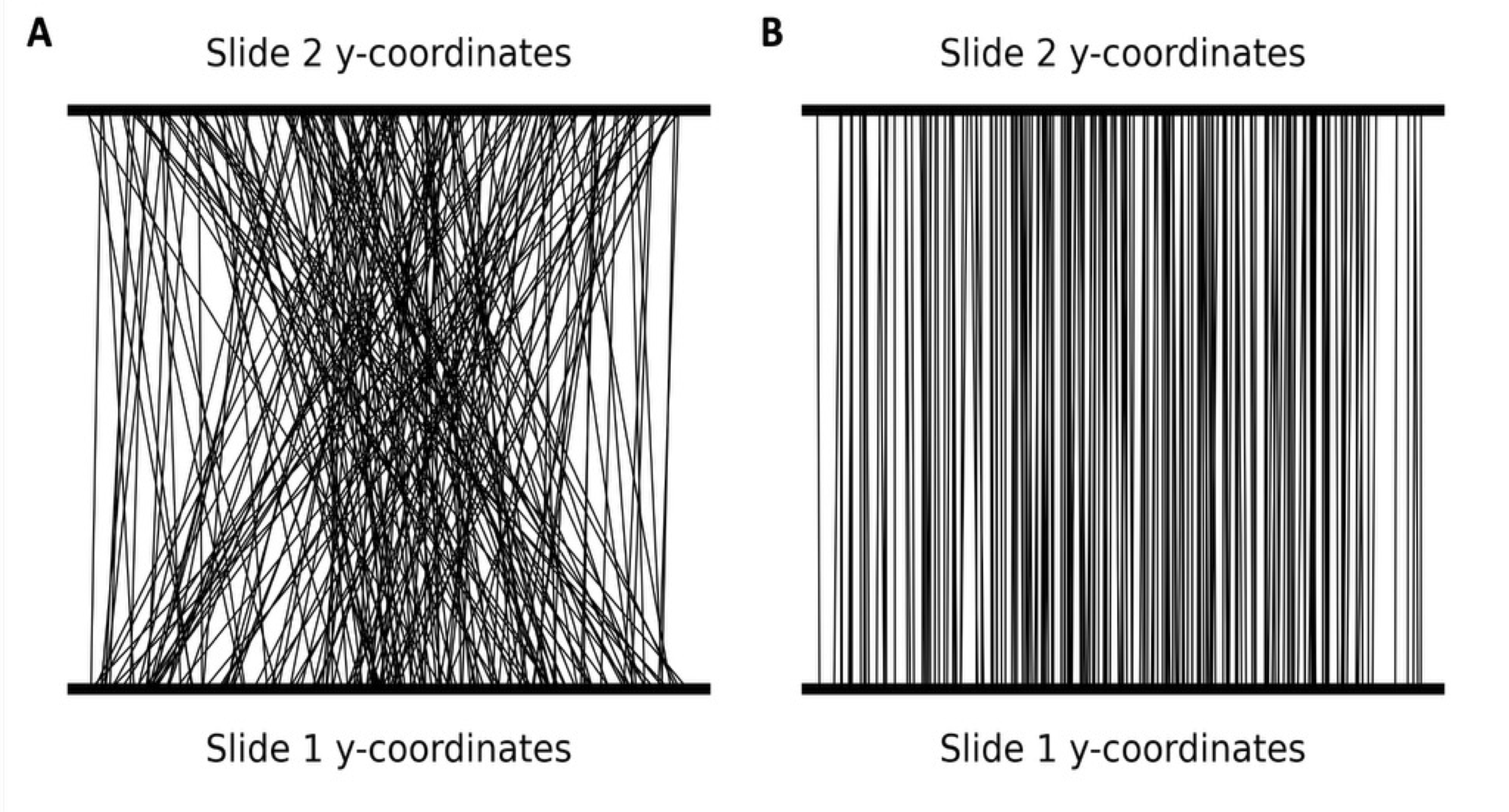

Supplement: S1 Fig — (A) y-coordinates of linear sum assignment matches connected by lines across Slide 1 and Slide 2 of core B3 shown on the y-z projection of serial slides. (B) y-coordinates of cell tracking matches connected by lines across Slide 1 and Slide 2 of core B3 shown on the y-z projection of serial slides. (TIFF) [file pcbi.1013325.s001.tiff]

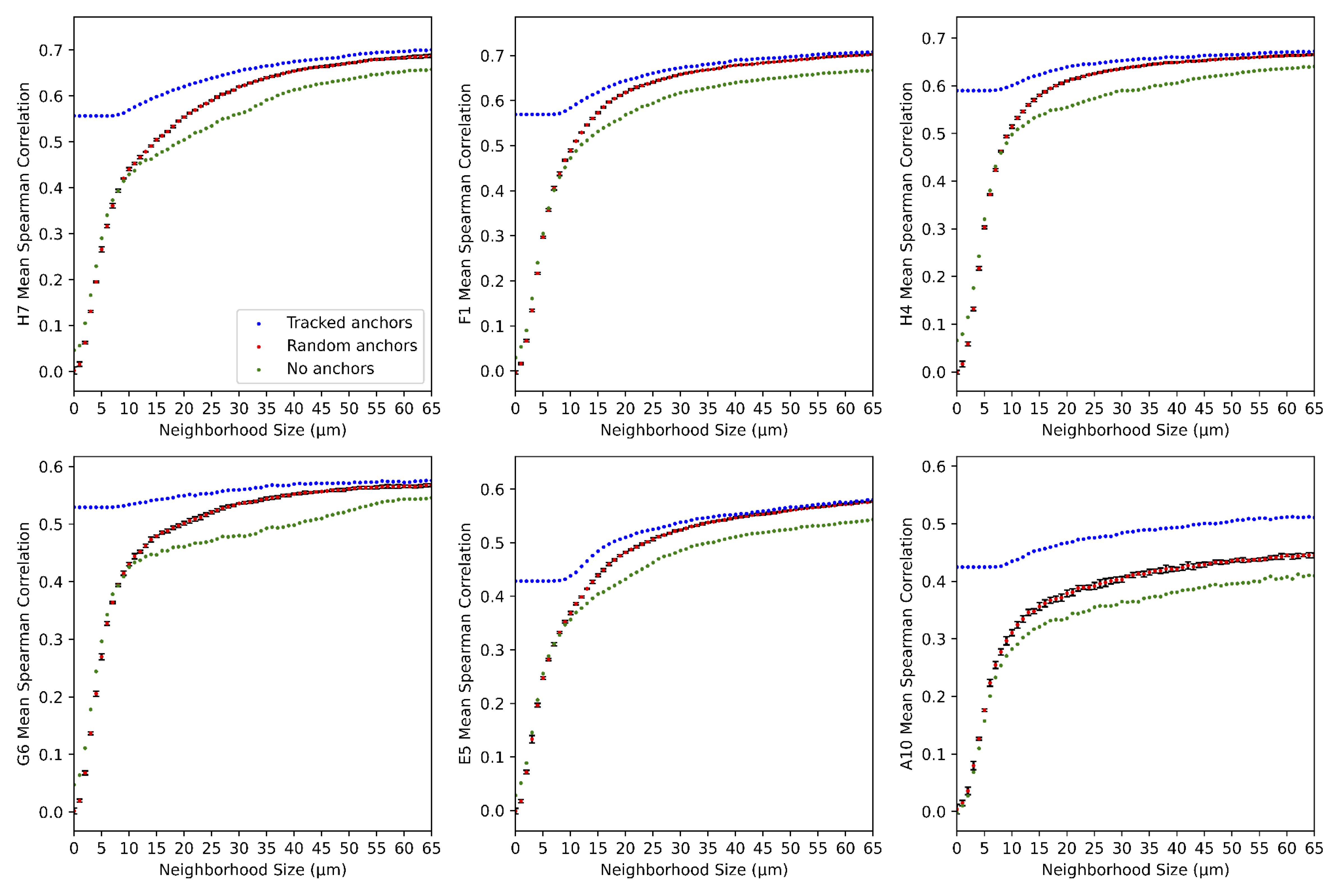

Supplement: S2 Fig — Shared marker correlation of cell pairs versus search radius for linear sum assignment matching based on tracked N anchors, random N anchors, and no anchors for cores H7, F1, H4, G6, E5, and A10. (TIFF) [file pcbi.1013325.s002.tiff]

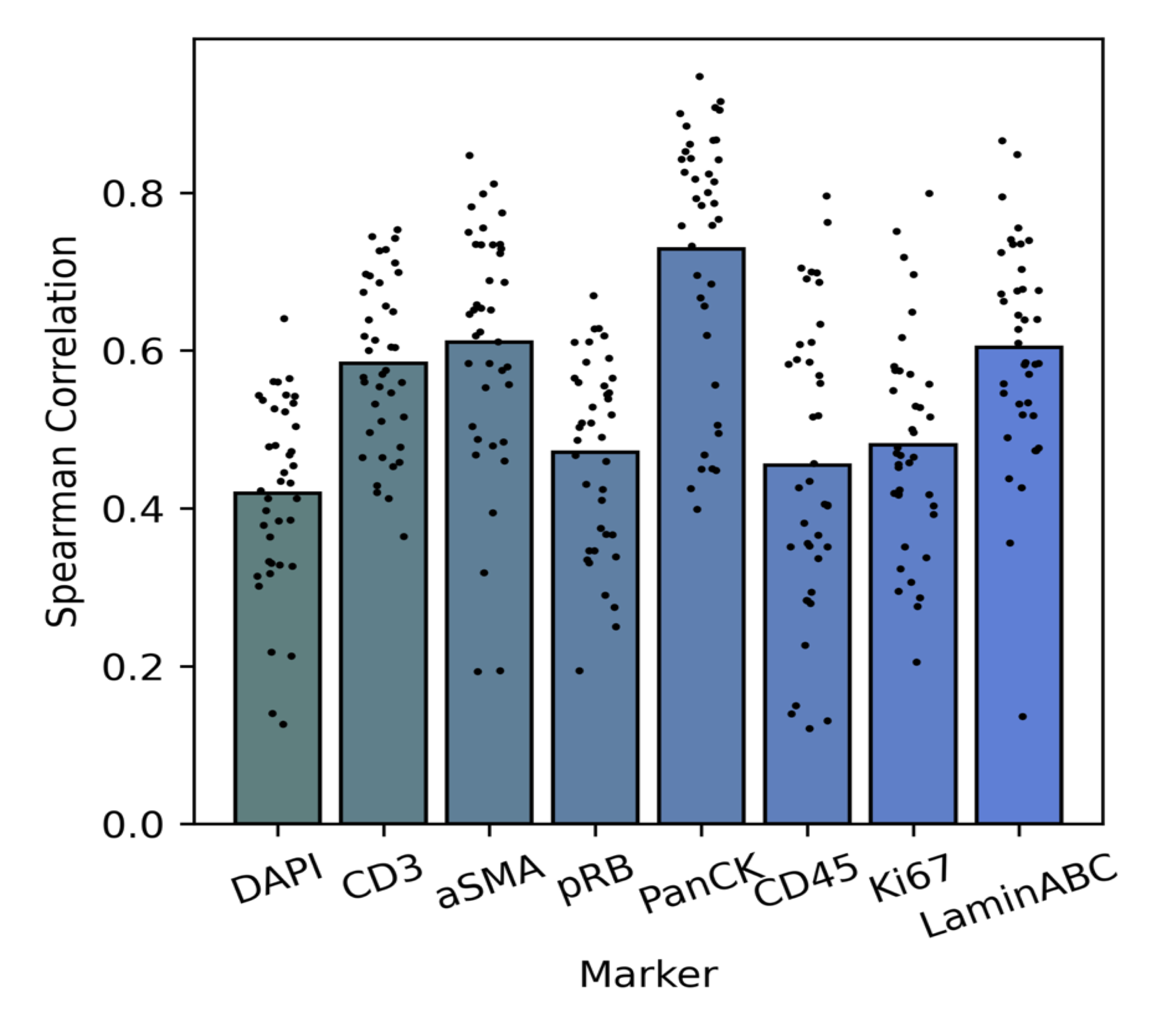

Supplement: S3 Fig — Mean Spearman correlation of 39 TMA cores for 8 shared markers. (TIFF) [file pcbi.1013325.s003.tiff]

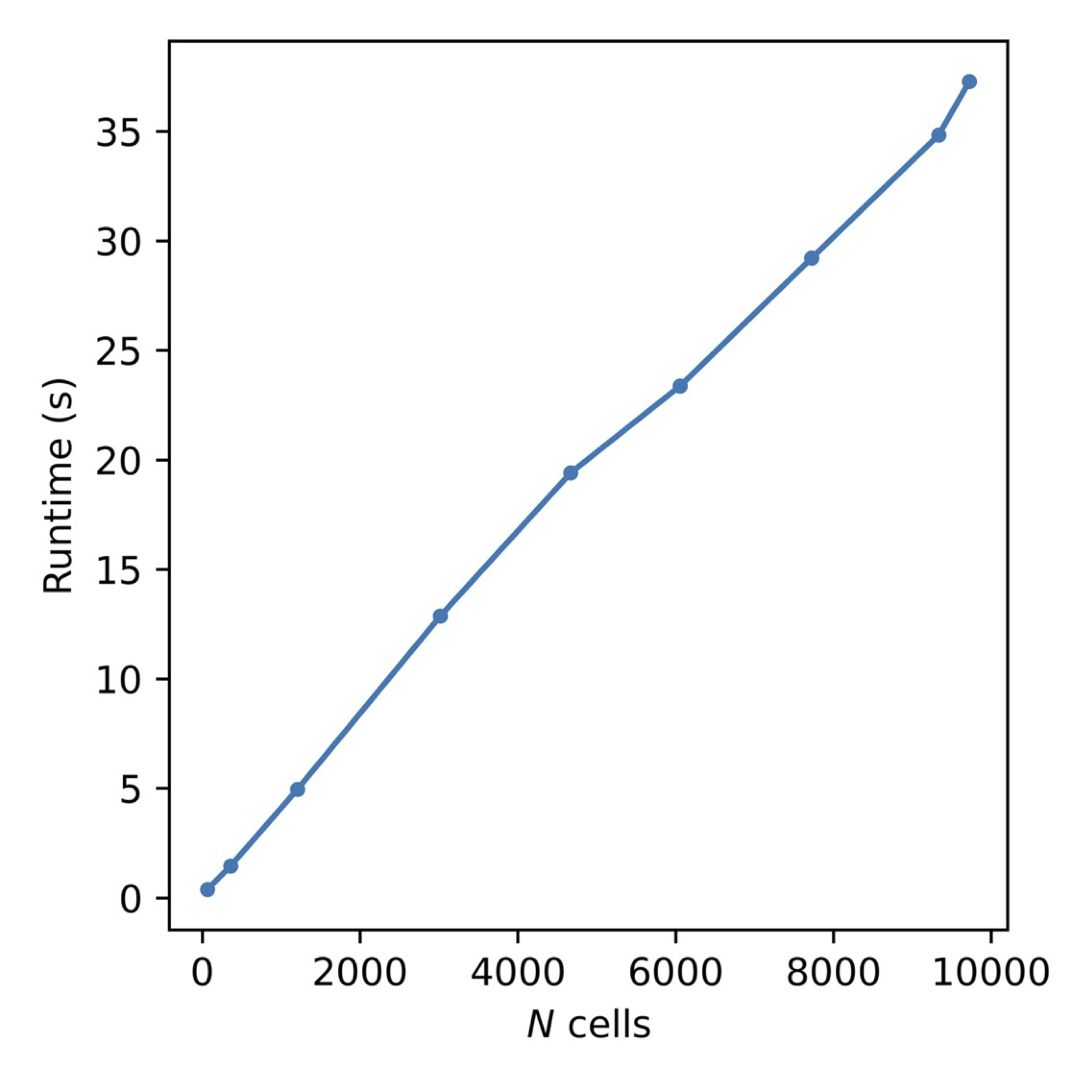

Supplement: S6 Fig — Runtime of the COEXIST algorithm with respect to the average number of input cells across consecutive MTIs using core B4 in the TNP-TMA dataset. (TIFF) [file pcbi.1013325.s006.tiff]

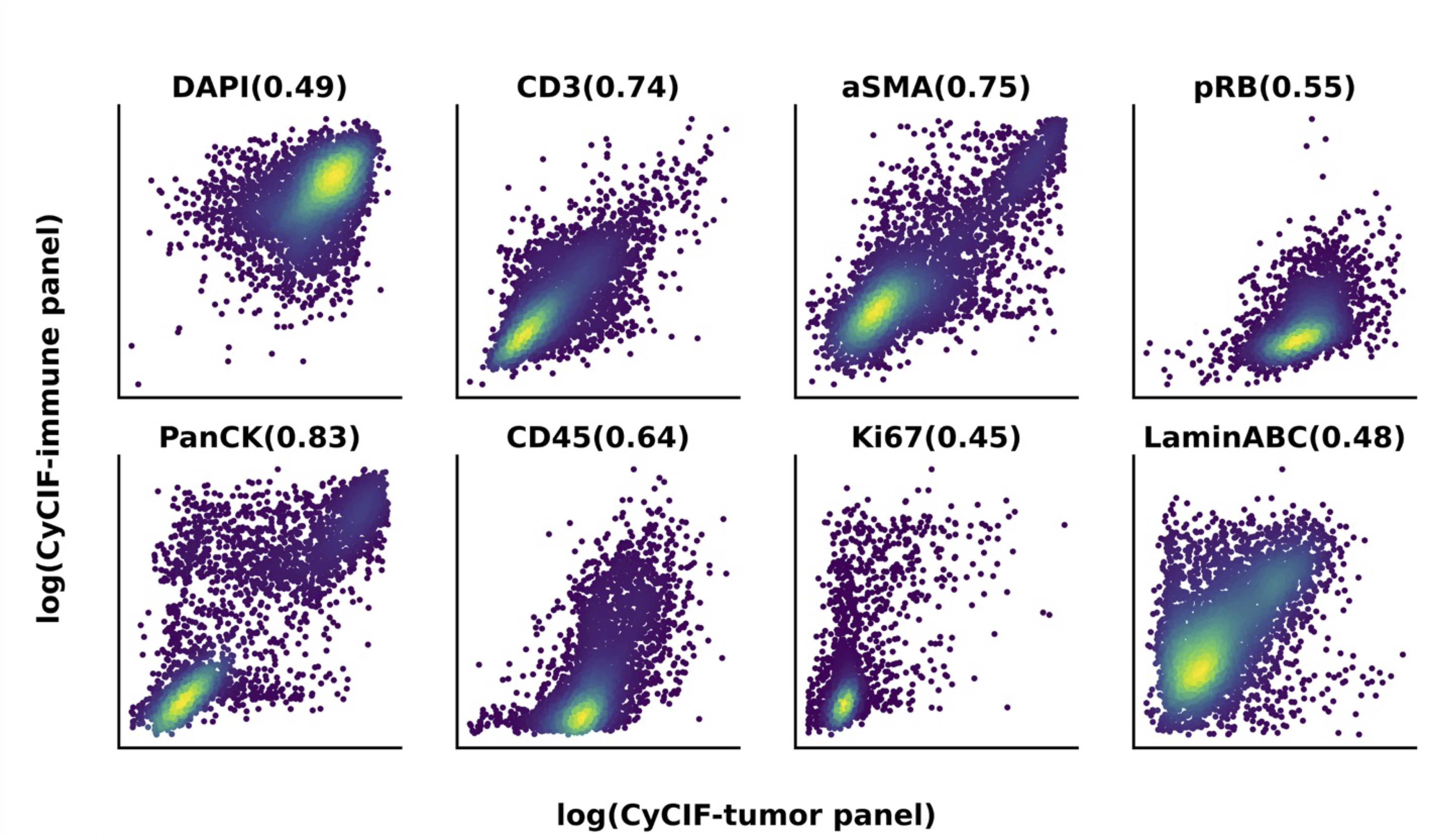

Supplement: S7 Fig — Single-cell scatter plots of marker intensity across shared markers in consecutive CyCIF MTIs for core B4. (TIFF) [file pcbi.1013325.s007.tiff]

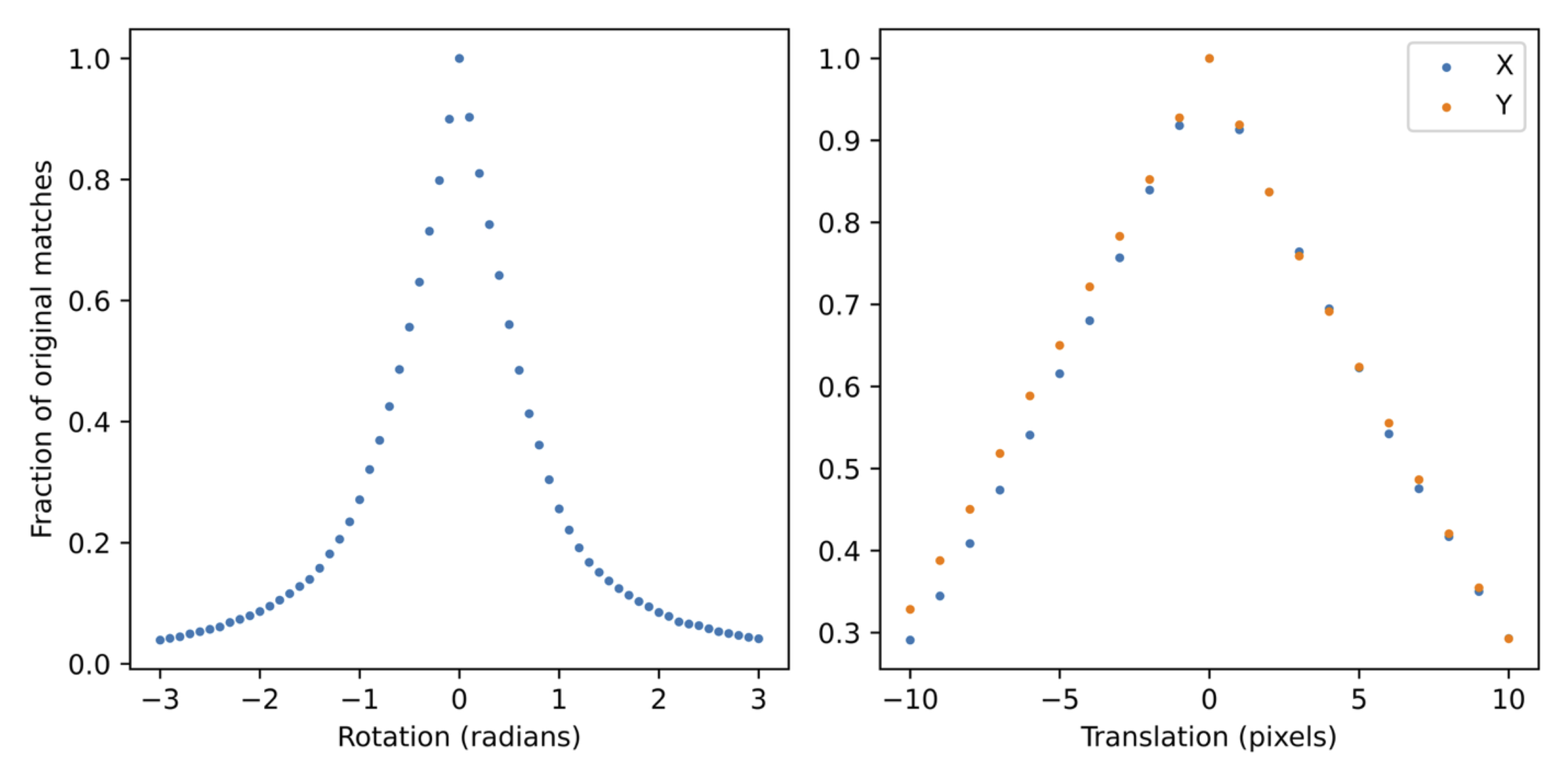

Supplement: S8 Fig — Match recovery after applying rotation and translation transforms to section 2 for core B4. (TIFF) [file pcbi.1013325.s008.tiff]
