# Supplementary material for: COEXIST: Coordinated single-cell integration of serial multiplexed tissue images
Source: PLoS Comput Biol. 2025 Aug 5;21(8):e1013325. doi: 10.1371/journal.pcbi.1013325 (PMC12338771; doi:10.1371/journal.pcbi.1013325)
Supplement: Attachment — Submitted filename: Rebuttal.docx [file pcbi.1013325.s011.docx]

Dear Editor and Reviewers,

On behalf of the authors, we would like to express our sincere gratitude for your time and effort in helping us improve our work. Below, we provide detailed responses to each reviewer’s comments, outlining how we have addressed the feedback in our revised manuscript. The updated manuscript is attached, with all revisions highlighted in yellow for easy reference.

Sincerely,

Young Hwan Chang, PhD

**Scientific Editor (Dr. Marc Birtwistle):**

Dear Dr. Chang,

Thank you for submitting your manuscript to PLOS Computational Biology. After careful consideration, we feel that it has merit but does not fully meet PLOS Computational Biology's publication criteria as it currently stands. Therefore, we invite you to submit a revised version of the manuscript that addresses the points raised during the review process.

Please submit your revised manuscript within 60 days Jul 29 2025 11:59PM. If you will need more time than this to complete your revisions, please reply to this message or contact the journal office at ploscompbiol@plos.org. When you're ready to submit your revision, log on to [https://www.editorialmanager.com/pcompbiol/](https://urldefense.com/v3/__https:/www.editorialmanager.com/pcompbiol/__;!!Mi0JBg!LbaYog_eIB7EQR7hr-SxgSN1p_WeXZzU13u_YqhtytfUKnyb5eBzofRd9izILaqIE-yzo3WtizPyUW8$) and select the 'Submissions Needing Revision' folder to locate your manuscript file.

Please include the following items when submitting your revised manuscript:

* A rebuttal letter that responds to each point raised by the editor and reviewer(s). You should upload this letter as a separate file labeled 'Response to Reviewers'. This file does not need to include responses to formatting updates and technical items listed in the 'Journal Requirements' section below.

* A marked-up copy of your manuscript that highlights changes made to the original version. You should upload this as a separate file labeled 'Revised Manuscript with Track Changes'.

* An unmarked version of your revised paper without tracked changes. You should upload this as a separate file labeled 'Manuscript'.

If you would like to make changes to your financial disclosure, competing interests statement, or data availability statement, please make these updates within the submission form at the time of resubmission. Guidelines for resubmitting your figure files are available below the reviewer comments at the end of this letter

We look forward to receiving your revised manuscript.

Kind regards,

Marc Birtwistle

Section Editor

PLOS Computational Biology

*Thank you for the opportunity to revise our manuscript and for sharing the insightful feedback from the reviewers. We sincerely appreciate the time and effort you and the reviewers have dedicated to evaluating our work.*

*We are grateful for your guidance and the opportunity to improve our work. Your support throughout this process has been invaluable, and we are committed to aligning the revised manuscript with the high standards of PLOS Computational Biology.*

*We look forward to your feedback on the revised submission.*

**Reviewer #1**: In this work, Heussner, Watson et al introduce COEXIST, a method for integration of cellular data across serially sectioned slides. This method shows great potential for improving multiplex integration for the many serial sectioning datasets emerging within the tissue atlas community. Below I provide detailed comments:

*Thank you for recognizing the potential of COEXIST to integrate consecutive MTIs.*

In the introduction, the authors write “to our knowledge, no algorithms or computational frameworks explicitly designed to pair single cells across consecutive tissue sections currently exist.” I advise avoiding statements claiming to be the first to do something, as this is hard to prove. As one counter example, I identified ref [1], a manuscript published in 1988 presenting a software solution for reconstruction of nuclei across serial electron microscopy images. More recent algorithms exist to do similar intra-slide cellular comparison.

*We acknowledge claims of being “the first” are difficult to prove and should be well-evidenced. In response, we removed this statement from the introduction and added the reference you provided to improve the contextual background.*

The authors determined that their dataset possessed a mean nuclear diameter of 7.8 micron, then used this diameter to simulate spherical nuclei for the cell tracking algorithm implementation. What are the consequences of this averaging for very small nuclei (such as lymphocytes) compared to very large nuclei (such as breast cancer cells). The error for these different cell types is likely very different. Lymphocytes may at times only exist in a single tissue section, while normal epithelial or cancer cells will nearly always exist in 2 – 3 sections. How does the accuracy of COEXIST vary across differently sized cell types?

*Thank you for your insightful comment regarding the impact of nuclear size on tracking accuracy.*

*To clarify, the simulation used the nuclear diameter statistics drawn from real MTIs to estimate the expected proportion of tracked cells across serial sections. At each simulation iteration, we draw nucleus diameter from a normal distribution parameterized by the real nuclear diameter distribution statistics (a mean of 7.8μm and a standard deviation of 2.4μm) across all tissue cores. Therefore, our simulation accounts for small cells in proportion to their prevalence in the tissue. However, as noted, both nuclear size and section thickness influence tracking success - smaller nuclei and thicker sections independently reduce the likelihood of a nucleus being present in multiple sections.*

*To examine this further, we conducted two supplementary experiments. First, we ran another simulation varying the mean nuclear diameter while fixing tissue section thickness 5μm and measured the theoretical tracking rate (Figure R1). Smaller nuclei (4μm) showed ~40% theoretical tracking, while large nuclei (10μm) reached ~65% theoretical tracking.*

***Figure R1****: Proportion of shared cells versus mean nuclear diameter distribution in Monte Carlo simulation.*

*Next, in the real dataset we took core B4 and compared nuclear diameters of all nuclei in section 1 to those of only the tracked nuclei in the same section (Figure R2). Shared cells had a slightly larger mean diameter (7.4μm versus all the cells 7.1μm), with a statistically significant difference (Kolmogorov-Smirnov statistic, comparing shared to all of 0.085 (p=9.9e-20)). However, the Pearson correlation of the histograms was high (Pearson r=0.99, p=5.5e-62), suggesting close overall similarity.*

***Figure R2****: Nuclear diameter distributions of all vs. tracked cells in one tissue section.*

*These experiments support your hypothesis that COEXIST’s matchings are sensitive to nuclear size. Importantly, while small cells are indeed less likely to be present in two adjacent sections due to their limited diameter, this does not necessarily degrade COEXIST’s performance. This reflects a fundamental imitation of physical sectioning rather than a failure of the algorithm. Even when individual cells are not tracked across sections, COEXIST relies on spatially stable anchor cells to propagate label information, allowing robust reconstruction of cell phenotypes and maintaining high-quality integration across sections. We acknowledge this limitation in our discussion on page 14, where we note:*

*“However, this assumption may not hold in cases where the tissue sections are thicker than the average cell diameter, potentially leading to a disproportionate representation of certain cell populations. For example, the immune cells, which tend to be significantly smaller than malignant cells, could be especially affected depending on how the section is cut in a 2.5-dimension.”*

*We appreciate your suggestion and agree this nuance is important for interpreting COEXIST’s applicability across diverse cell populations.*

In the methods section, the authors write they removed 49 of 88 TMA cores from their dataset due to imaging artifacts, tissue loss, registration failure, or non-consecutive tissue sections to evaluate COEXIST only on very high-quality data. However, implementation of this algorithm by others will necessitate its application on non-ideal datasets. The authors should analyze a small number of non-perfect serial images (non-consecutive, imperfectly registered, etc.) to quantify the loss in accuracy compared to higher quality datasets. Alternatively, the authors could use their simulation framework to iteratively reduce the quality of their virtual dataset and compute the loss in accuracy here. In the discussion section, the authors should provide further detail regarding the technical limitations of this software in non-perfect serial datasets.

*We agree that non-ideal datasets are common in multiplex tissue imaging but emphasize that high-quality registration is a prerequisite for applying COEXIST. Prior to running COEXIST, users need to check and filter their datasets to regions of high registration quality, similar to how we filtered the TMA dataset to high-quality cores. These types of quality requirements are common for registration tools such as VALIS [2] and ASHLAR [3]. Both tools explicitly state that severe tissue deformations or imaging artifacts may cause decreased registration quality. Similarly, COEXIST assumes reliable alignment; poor registration will directly reduce the number of trackable cells and introduce errors in matching.*

*Furthermore, COEXIST relies on shared marker proteins for both integration and cell type propagation. If a marker exhibits non-specific staining, signal dropout, or illumination artifacts on either section, this can lead to spurious cell matching or incorrect label transfer. Therefore, users must ensure that shared marker channels are of sufficient quality across both sections before applying COEXIST.*

*In response to this concern, we have added language to the manuscript emphasizing the necessity of users to perform quality control checks and sample filtering prior to applying COEXIST.*

*Regarding robustness to degraded input, the primary downstream effect of registration error, tissue loss, or other artifacts is a reduction in the proportion of cells that can be reliably tracked across sections. In Figure 3, our simulation explores a wide range (0.1-0.9) of trackable cell proportions, which implicitly models varying degrees of data quality. The lower end of this range corresponds to datasets with more severe artifacts or lower overlap, and the resulting accuracy loss on cell-type propagation is demonstrated in our existing simulation across this range.*

*Additionally, we performed a new experiment to quantify COEXIST’s tolerance to registration errors. We introduced synthetic misalignments - rotation and translation - into registered images and measured the fraction of original ground-truth matches recovered by COEXIST. We found that COEXIST remains relatively robust to small perturbations (rotations of +/-0.2 radians or translations of +/-3 pixels in either X or Y), recovering approximately 80% of the original matches (Figure R3). We added this figure to the manuscript as supplementary figure 8.*

***Figure R3****: COEXIST’s robustness to registration perturbations.*

*In summary, COEXIST assumes a reasonably well-registered input dataset and performs best under these conditions. We recommended that users assess registration quality before application and refer to the Monte Carlo simulation results as a guide to expected performance based on tractable cell fractions. When datasets fall outside this expected range, further investigation into registration accuracy is warranted.*

References:

[1] Geraud et al, “Three-dimensional computer reconstructions from serial sections of cell nuclei”, Biology of the Cell, 1988

[2] Gatenbee, C.D., Baker, AM., Prabhakaran, S. et al. Virtual alignment of pathology image series for multi-gigapixel whole slide images. Nat Commun 14, 4502 (2023).

[3] Jeremy L Muhlich, Yu-An Chen, Clarence Yapp, Douglas Russell, Sandro Santagata, Peter K Sorger, Stitching and registering highly multiplexed whole-slide images of tissues and tumors using ASHLAR, Bioinformatics, Volume 38, Issue 19, October 2022, Pages 4613–4621

**Reviewer #2**: The authors present a novel method for linking molecular profiles across slices in multiplexed tissue imaging, addressing the challenge of incomplete molecular profiles and potential biases in cell type phenotyping due to tissue sectioning and low mRNA counts. While previous approaches used molecular profiles to match cells across slices via a linear assignment problem, the authors compellingly argue that such methods can artificially reduce molecular profile diversity by enforcing cell consistency. Instead, their proposed COEXIST method links cells by minimizing the 1-Spearman correlation coefficient of overlapping cells' molecular profiles, thereby removing the need for predefined maximum distances or anchors. The approach is validated through a didactic Monte-Carlo simulation and tested on several publicly available datasets, which is commendable.

*Thank you for accurately summarizing our work.*

I had some difficulty fully grasping the COEXIST method solely from the manuscript without referring to the provided code. Specifically, the integration of spatial localization and molecular profiles, which draws upon concepts from cell tracking and linear sum assignment discussed in preceding sections of the Results, could be further clarified. The method's presentation, while concise, leaves a few points ambiguous. I recommend briefly reiterating the key steps or including an algorithm pseudocode to enhance clarity. For instance, on page 20, the statement "We use D as described previously in place of the distance measure (region overlap or centroid distance) in the cell tracking algorithm. Therefore, spatially coherent candidate cells are considered, but matches are assigned by shared molecular profiles" might be less confusing if rephrased. Perhaps something like: "Across a set of spatially overlapping cells, matching cells are defined by minimizing the 1-Spearman correlation coefficient D between their molecular profiles," assuming this interpretation is correct.

*Thank you for this helpful suggestion. We agree that the methods section could be clearer, particularly in explaining how spatial localization and molecular profiles are integrated in COEXIST. As you noted, the description relied on earlier concepts from cell tracking and linear sum assignment, but the concise presentation may leave some steps ambiguous.*

*In response, we have revised the relevant paragraph to improve clarity. Additionally, we have added a COEXIST pseudocode on page 21 to help clarify the algorithm to the reader. We hope these changes make the method more accessible without requiring direct reference to the code.*

On page 8, the authors note that 'LSA, ... can yield unstable results depending on the order of reference.' Could the authors elaborate on whether the approach would exhibit order invariance if the cost matrix were symmetric?

*Thank you for noting this important point. If the cost matrix were symmetric, the linear sum assignment would exhibit order invariance. However, in practice, serial sections rarely contain the same number of cells, and arbitrarily excluding unmatched cells on one side can lead to unstable or inconsistent pairings. We have clarified in the manuscript that the observed instability stems from the asymmetry of the cost matrix and the mismatch in cell counts across sections.*

Page 17 mentions the removal of nonconsecutive slices during quality control. It would be valuable to understand the sensitivity of the proposed approach to registration errors between consecutive slices. Inaccuracies in cell registration could potentially invalidate the tracking approach. I suggest the authors consider adding an experiment to investigate the impact of such registration errors.

*Thank you for this important point. As noted in our response to Reviewer 1, we agree that COEXIST’s performance is sensitive to registration accuracy and have added an experiment to evaluate its robustness under controlled registration errors.*

The cell segmentation on page 17 is performed using the mesmer cell segmentation method [45]. Given that mesmer can also perform 3D segmentation by linking regions across sections, it would be beneficial if the authors could discuss this alternative approach within the text.

*This is a fair point. 3D segmentation options for Mesmer and Cellpose were designed for and tested on thin tissue sections (2-3µm) in which nuclei are more easily preserved across adjacent sections. Additionally, Cellpose 3D segmentation uses a fixed intersection over union (IoU) threshold to consider a cell tracked across adjacent sections. COEXIST has two primary innovations which extend and complement existing 3D segmentation tools: 1) COEXIST leverages biomarker information to match cells across adjacent sections, and 2) COEXIST uses a radius to restrict possible cell matches which is more flexible than a simple overlap threshold which could miss possible cell matches, especially over larger tissue sections (5µm).*

*These features make COEXIST particularly well-suited for tissue samples not specifically prepared for 3D segmentation. That said, COEXIST is also compatible with 3D segmentation tools like Mesmer or Cellpose, to refine cell tracking or propagate cell-type labels to untracked cells using molecular context.*

*We have added this discussion to the manuscript to clarify how COEXIST complements existing segmentation tools and to help guide users working with non-ideal or thicker tissue samples.*

On page 18, I was unable to identify the principle of Voronoi partitioning via stable matching [36] in the described tracking approach. The current method appears to match cells based on the maximum overlap between nuclear masks, which seems unrelated to algorithm (1) in reference [36]. As there is no apparent Voronoi partitioning or stable matching algorithm employed in the text or code, I suggest reviewing or removing this reference.

*Thank you for pointing this out. Upon further review, we agree that the tracking algorithm we implemented does not directly employ Voronoi partitioning via stable matching, as described in the aforementioned reference [36]. We have revised the manuscript to remove the citation at this location and clarify this accordingly.*

Regarding the notation on page 18, in 'P ∩ Q defined as subsets P ⊆ P and Q ⊆ Q', it appears that P and Q are subsets of themselves by definition. Could the authors clarify this notation?

*Thank you for pointing this out. The notation in question may be confusing because the subsets P and Q are supposed to be italicized. To improve clarity, we have edited the text to refer to these subsets as* A *and* B *and updated the relevant sections accordingly.*

Also on page 18, could the authors please clarify the meaning of the notation n(P)=n(Q)?

*Yes, we clarify n(X) refers to the number of elements in X. The notation n(P)=n(Q) (now n(*A*)=n(*B*)) indicates that subsets P and Q (*A *and* B*) have the same cardinality.*

Finally, on page 21, the decision to perform clustering after cell matching is highly relevant and effectively showcases the utility of the cell matching. However, it's not entirely clear why clusters are computed solely on cells shared across slices, rather than considering all cells for cluster definition. Further clarification on this choice would be appreciated.

*Thank you for highlighting this point. We chose to perform clustering only on the shared cells to take advantage of the expanded marker panel available through cell matching, enabling higher-resolution and more robust cell type classification. This approach is illustrated in Figure 4B, which demonstrates how cell type assignments improve when using the combined marker information from tracked cells, compared to clustering based on either section alone.*

*Additionally, in many practical MTI experiments, consecutive sections may contain limited overlap in markers or may be optimized for distinct biological targets. By clustering only the matched cells, we ensure that the clustering reflects the full combined marker set without introducing inconsistencies from partially overlapping profiles. We have added this explanation to the manuscript for clarity.*
